# Echinococcal cyst of the left vas deferens – a case report and literature review

**Published:** 2014

**Authors:** CP Farcaş, A Rădulescu, M Dinu, V Mădan, O Bratu, D Spînu, R Popescu, D Mischianu

**Affiliations:** * “Carol Davila” University of Medicine and Pharmacy, Bucharest, Romania

**Keywords:** cyst, parasite, vas deferens, excision

## Abstract

Cystic echinococcosis or hydatid disease is an important public health issue, mainly in developing countries, due to its high prevalence. Echinococcus granulosus, a cyclophyllid cestode, the pathogenic parasite found in humans, their intermediate host in its way to the final host, the members of canidae family.

The main sites of infection in humans are the liver and the lungs. There have been recorded Rare locations such as the heart, spleen, muscles or retroperitoneal have also been recorded.

**Case presentation:** We present the case of a 29-year-old man, living in rural environment, who was admitted in our Clinic for a pelvic cystic tumor and intermittent ureterohydronephrosis. The blood work showed positive antibodies for Echinococcus granulosus. An urethrocystoscopy and the excision of the tumor were performed.

**Conclusions:** Although rare, the involvement of the male genitourinary tract in cystic echinococcosis is possible and can pose important diagnostic challenges.

## Introduction

Cystic echinococcosis is a parasitic disease, a real public health issue [**1**], caused by tape worms from the genus Echinococcus. The disease is provoked by the larval stage of Echinococcus granulosus, Echinococcus multilocularis – with an alveolae like pattern of growth, and very rare Echinococcus vogeli causing polycystic echinococcosis.

Even if it is one of the oldest diseases acknowledged by humankind, the Talmud having mentioned it and Hippocrates having recognizing it, cystic echinococcosis is an important public health issue being endemic in some regions of the world. 

The parasite’s life cycle has an intermediate host before infecting its final host, belonging to the canidae family, mainly dogs but also wolves, dingoes and other carnivores. The humans act as an accidental intermediate host when handling products infected with parasite eggs. The disease is more common in pastoral regions but it is not unusual in urban settlements [**2**].

From its digestive port of entry, the parasite passes into the portal circulation and then passes through three capillary filters: liver, lung and systemic circulation. Then it can virtually reach any organ, where it develops into cysts.

The cystic lesion has three layers: the pericyst, the laminated membrane and the endocyst, which produces the infectious solices.

The main sites of infection are the liver and the lungs, recording more than 90% of all cases. Other sites such as the genitourinary tract are uncommon [**4**].

The clinic of Cystic echinococcosis can be poor for a long period of time following the infection due to slow development rate. Symptoms may vary from jaundice to anaphylaxis [**4**,**5**].

Diagnosing the disease must combine imagistic findings with serological tests.

The treatment for the localized disease is multimodal and mainly consists of a surgery in association with chemotherapy with albendazole [**6**]. In spite of incomplete notification of cases, Romania is considered a highly endemic territory [**2**].

## Case presentation

N.N., a 29-year-old male, living in rural environment was admitted in our Clinic for a pelvic retrovesical cystic tumor, intermittent ureterohydronephrosis, dysuria and mild lumbar pain.

The patient had no significant medical history and lived in rural environment, frequently handling domestic animals.

**Fig. 1:**
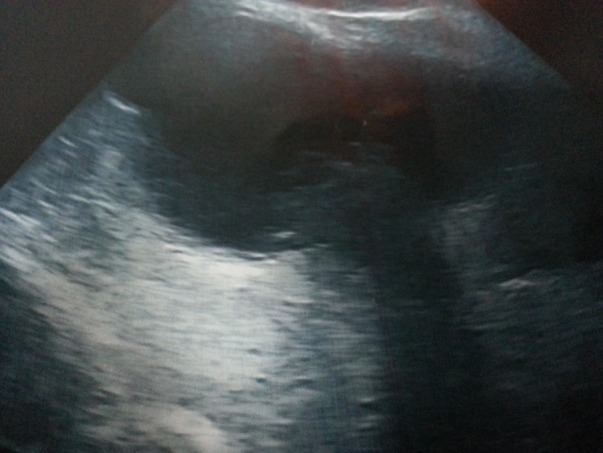
Ultrasonographic aspect of the paravesical cystic lesions

The abdominal ultrasound showed normal liver, kidneys, enlarged spleen and a homogenous prostate with a volume of 25 cubic centimeters. The urinary bladder had regular walls. Laterally, on the left side of the bladder, several cystic lesions were described, the largest measuring 72/106 millimeters (**Fig. 1**).

The patient underwent a semen analysis, which showed important oligospermia, asthenozoospermia and teratozoospermia.

**Fig. 2:**
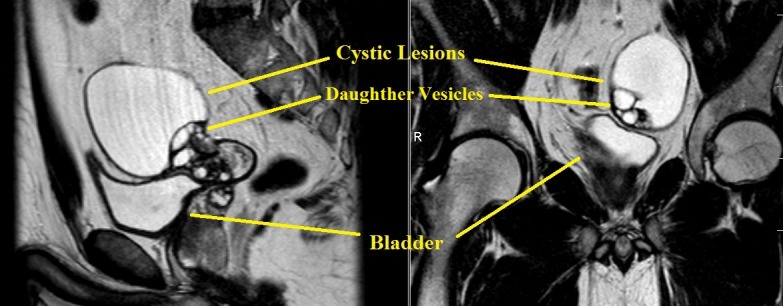
MRI aspect of the cystic lesions

A pelvic MRI scan showed a large cystic lesion measuring 85/67 mm axially and 75 mm cranio-caudally with adjacent smaller multicystic lesions situated superiorly and laterally of the bladder - daughter vesicles (**Fig. 2**). The left paravesical cystic lesions had no pathologic contrast enhancement.

The prostate was homogenous and had a volume of 23 cubic centimeters with no pathological elements. Several enlarged inguinal lymph nodes were present.

The patient tested positive antibodies for Echinococcus granulosus. The patient was referred to a parasitologist who confirmed the diagnosis and recommended chemotherapy with albendazole prior and after surgery.

Combing the imagistic, clinic and serological findings, the diagnosis was set as left vas deferens hydatid cyst. The decision was to perform a surgical treatment after several doses of albendazole. 

**Fig. 3:**
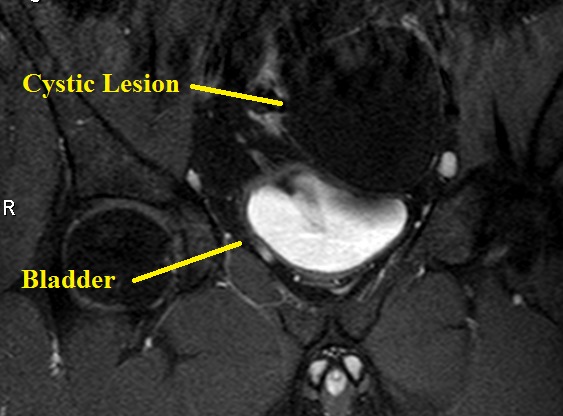
No contrast enhancement in the cystic lesions

The hydatid cysts were excised, under general anesthesia and by means of subumbilical incision, as a whole with extreme caution in order not to spread the daughter vesicles. The integrity of the vas deferens and the left seminal vesicle was maintained. The patient was set on intense antiparasitic treatment, with albendazole, as recommended by the parasitologist. Five days after the surgery, the patient was discharged with a good general status and satisfactory voiding.

**Fig. 4:**
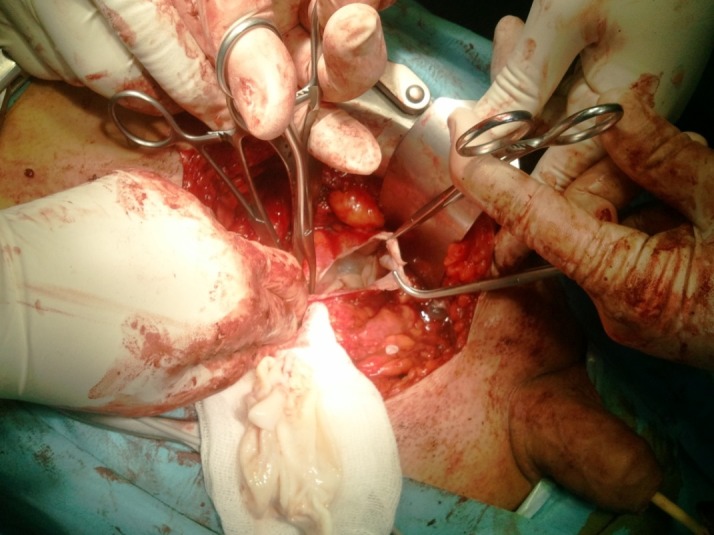
Intraoperative aspect of the cystic lesion with daughter vesicles

At three months, no dysuria was present, no left urinary obstruction appeared at the intravenous urography and the semen analysis was improved.

**Fig. 5:**
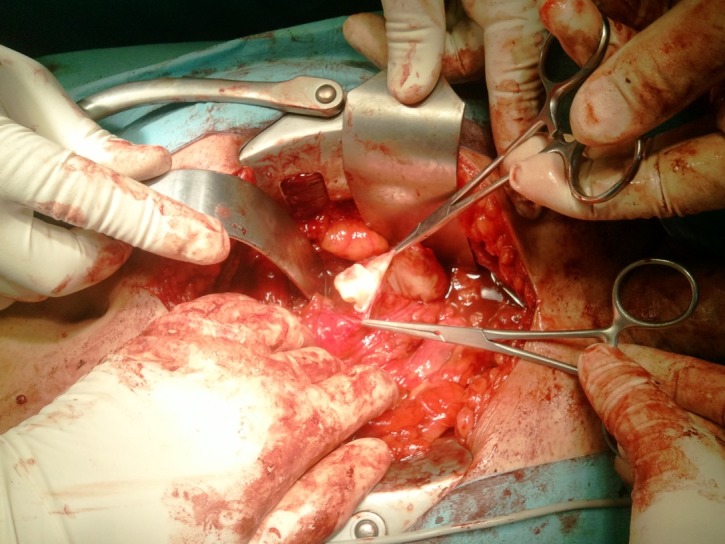
Surgical extraction of a daughter vesicle

## Discussion

Although rare, vas deferens and seminal vesicles may become sites for echinococcus infection. As our case showed, surgery combined with chemotherapy may cure the symptoms and prevent daughter vesicles from spreading and finally curing this parasitic disease, which by no means is declining in terms of prevalence.

The urologist should be aware of the possibility of dealing with a hydatid disease in the urogenital apparatus and should know that surgery and chemotherapy can almost or even entirely restore the function of the affected organ, as it happened in the case presented.
